# A Simulation Toolkit for Testing the Sensitivity and Accuracy of Corticometry Pipelines

**DOI:** 10.3389/fninf.2021.665560

**Published:** 2021-07-26

**Authors:** Mona OmidYeganeh, Najmeh Khalili-Mahani, Patrick Bermudez, Alison Ross, Claude Lepage, Robert D. Vincent, S. Jeon, Lindsay B. Lewis, S. Das, Alex P. Zijdenbos, Pierre Rioux, Reza Adalat, Matthijs C. Van Eede, Alan C. Evans

**Affiliations:** ^1^McGill Centre for Integrative Neuroscience, Montreal Neurological Institute, Montreal, QC, Canada; ^2^PERFORM Centre, Concordia University, Montreal, QC, Canada; ^3^Sick Kids Research Institute, Toronto, ON, Canada

**Keywords:** reproducible neuroimaging, cortical thickness, lesion simulation, pipeline accuracy, brain morphometry, statistical parametric mapping

## Abstract

In recent years, the replicability of neuroimaging findings has become an important concern to the research community. Neuroimaging pipelines consist of myriad numerical procedures, which can have a cumulative effect on the accuracy of findings. To address this problem, we propose a method for simulating artificial lesions in the brain in order to estimate the sensitivity and specificity of lesion detection, using different automated corticometry pipelines. We have applied this method to different versions of two widely used neuroimaging pipelines (CIVET and FreeSurfer), in terms of coefficients of variation; sensitivity and specificity of detecting lesions in 4 different regions of interest in the cortex, while introducing variations to the lesion size, the blurring kernel used prior to statistical analyses, and different thickness metrics (in CIVET). These variations are tested in a between-subject design (in two random groups, with and without lesions, using T1-weigted MRIs of 152 individuals from the International Consortium of Brain Mapping (ICBM) dataset) and in a within-subject pre-/post-lesion design [using 21 T1-Weighted MRIs of a single adult individual, scanned in the Infant Brain Imaging Study (IBIS)]. The simulation method is sensitive to partial volume effect and lesion size. Comparisons between pipelines illustrate the ability of this method to uncover differences in sensitivity and specificity of lesion detection. We propose that this method be adopted in the workflow of software development and release.

## Introduction

Morphometric neuroimaging pipelines are widely used to study human brain development and diseases. Cortical thickness, estimated from human magnetic resonance images (MRIs), is one such metric commonly used in brain development/degeneration studies (Thompson et al., 2003; Dickerson and Sperling, 2005; Lerch et al., 2005; Han et al., 2006). Several automated cortical-thickness measurement methods have been developed, such as ANTs (Tustison et al., 2010, 2014), CIVET (MacDonald et al., 2000; Kim et al., 2005), FreeSurfer (Dale and Sereno, 1993; Dale et al., 1999; Fischl and Dale, 2000; Fischl, 2012), and LOGIMBOS (Oguz and Sonka, 2014; Oguz et al., 2015).

Concerns arising from lack of reproducibility observed across different operating systems (Gronenschild et al., 2012; Glatard et al., 2015), necessitate to develop benchmarking methods to trace spurious variations, introduced by computational rather than biological variations, in the statistical outcomes of neuroimaging studies.

Morphometric pipelines apply several image processing methods to define the shape and boundaries of the white- and gray-matter surfaces, in order to optimally tessellate them, and link the surfaces in biologically plausible ways (Lerch et al., 2005; Tustison et al., 2010; Fischl, 2012; Oguz and Sonka, 2014). As such, these methods are highly sensitive to computational errors arising from parametrization (for example, thresholds of pre-processing parameters that determine the degree of noise reduction or tissue classification).

To ensure the validity of these pipelines, some have compared the validity of automated brain segmentation against manual segmentation of MRIs (Kabani et al., 2001; Kuperberg et al., 2003). Others have compared the validity of surface extraction against histological data (Rosas et al., 2002; Cardinale et al., 2014). However, given that MRI scans used in neuroimaging studies have highly variable quality and that inter-rater reliability is often low, manual segmentations are not the best approach to validation of such pipelines.

Morphometric pipelines are rarely used for absolute quantification of the cortical thickness (unless in cases where cortical pathology is expected, such as malformations of cortical development). Rather, they are used for uncovering the regions that may be commonly affected by neurodegenertation or atypical neurodevelopment, at the population level.

To evaluate the performance of different pipelines, Redolfi et al. (2015) compared two popular pipelines (CIVET 1.1.9 and FreeSurfer 5.3). They examined a statistical mapping of the correlations between cortical thickness and MMSE (Mini Mental State Examination) score in relation to the progression of Alzheimer’s disease. Han et al. (2006) used test-retest datasets, both within and across scanner platforms, with different sequence types and field strengths to show that thickness estimates with FreeSurfer were reliable when processing methods and MRI acquisition parameters were held constant. Another study concluded that FreeSurfer was highly reliable in a healthy elderly population (Liem et al., 2015). While valuable, these methods are limited in their ability to establish the validity of a given pipeline against the ground truth, and do not provide quantitative metrics to iteratively examine the sensitivity of different pipelines to different experimental factors.

An alternative approach to test pipeline validity against the “ground truth” is to simulate changes in the cortical thickness, and then evaluate the accuracy of detected change against the induced lesion. Lerch and Evans were the first to explore this method to evaluate the accuracy of different cortical thickness metrics, and the impact of blurring kernels, and sample size on the detection power of an early version of the CIVET pipeline (Lerch et al., 2005). One limitation of their study was that the size of the lesion was at voxel scale and that the location of the lesion was fixed. Later, Van Eede et al. (2013) have presented a simulation framework in rodents that allows to modify the shape of an anatomical structure at sub-voxel scales by defining a region of interest and a tolerance area within which the tissue can be shrunk or expanded in any direction.

In this study, we have adapted their method to apply small and simple lesions in the cortex. In this report we aim to present a simple case study using this approach to illustrate its application in comparing the sensitivity and specificity of lesion detection in different versions of the same pipelines, in relation to lesion location and size.

## Materials and Methods

### Experimental Design

**FIGURE 1:**
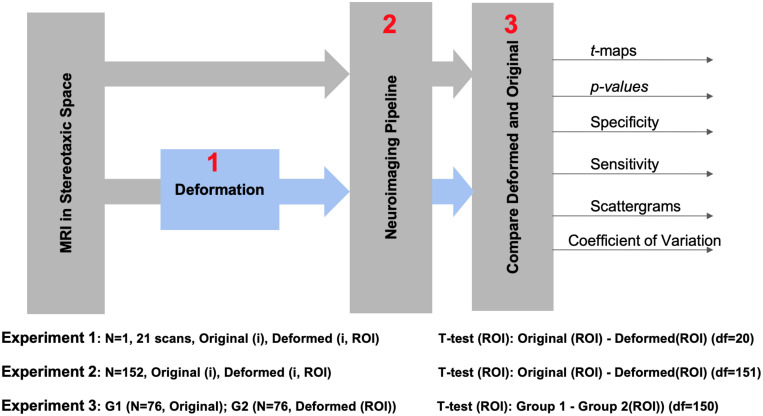
The flowchart of the implemented statistical analyses.

Figure 1 illustrates the experimental design of this study. In order to minimize variations arising from registration, we registered all raw data into the MNI152 stereotaxic space. We performed three experiments: to evaluate different pipelines in the absence of anatomical variations (Experiment 1); in presence of anatomical variations across the population, when the same subject was studied under two conditions (Experiment 2); in comparison of two independent groups (Experiment 3). Dependent and independent variables and test outcomes are summarized in Table 1.

**TABLE 1:** The outputs of the Simulation tool.

CoV	Coefficient of Variations (for Within Subject Tests)
***T*-Maps**	Calculated *T*-values all over the brain surface.
***P*-Values**	Calculated *P*-values all over the brain surface.
**Sensitivity**	True positive rate.
**Specificity**	True Negative rate.
**Scattergrams**	Shows *T*-values of the vertices against the distance to the deformed center.

### Datasets

Experiment 1 used the IBIS-Phantom: A subset of 21 T1-weighted MRIs of the same adult male subject brain from the Infant Brain Imaging Study (IBIS-Phantom)^1^ were randomly selected from over 100 available scans. All were acquired on 3T scanners with the same acquisition protocol. These images are used for the within-subject analysis to test for reproducibility under conditions of minimal anatomical variability.

Experiments 2 and 3 used the International Consortium for Brain Mapping (ICBM) data set: 152 MRIs collected at the MNI from healthy young adults (age 25–40) on a single Philips 1.5T scanner at a 1 mm isotropic resolution (Mazziotta et al., 2001)^2^. These images serve the population-level analysis in our study (both independent and dependent tests) and offer a more realistic assessment of detection power. More information about the scans are shown in Table 2. In Experiment 3, we selected a random sample of 76 samples from the MNI152 dataset (already in stereotaxic space), to deform at stereotaxic ROIs.

**TABLE 2:** The information about the ICBM scans.

			Unaltered subsample		Deformed subsample	
	ICBM scans (*N* = 152) Age *mean ± std:* 25.05 ± 4.79		ICBM scans (*N* = 76) Age *mean ± std:* 25.43 ± 5.50		ICBM scans (*N* = 76) Age *mean ± std:* 24.66 ± 3.95	
Gender	Female	Male	Female	Male	Female	Male
	*n* = 67	*n* = 85	*n* = 32	*n* = 44	*n* = 35	*n* = 41
Age mean ± std	24.68 ± 4.55	25.61 ± 4.90	24.12 ± 4.91	26.38 ± 5.76	24.51 ± 4.32	24.78 ± 3.66

**Thickness calculated for unaltered scans *mean ± std***						

	**ICBM scans (*N* = 152)**		**ICBM scans (*N* = 76) Group-1**		**ICBM scans (*N* = 76) Group-2**	

*CIVET2.1.1 tlaplace*	3.27 ± 0.53 (mm)		3.27 ± 0.53 (mm)		3.27 ± 0.48 (mm)	
*FreeSurfer 6.0*	2.43 ± 0.53 (mm)		2.43 ± 0.53 (mm)		2.43 ± 0.52 (mm)	

### Independent Variables

Table 3 shows the variables used in this work.

**TABLE 3:** The variables used in the analyses.

Dependent variables	Independent variables
Coefficients of Variation (CoV)	Regions of Interest (ROI)
Statistical Parametric Mapping	Thickness Metrics
Specificity	Blurring Kernels
Sensitivity	Neuroimaging Pipelines
*T*-Value Scattergrams	Amount of deformation
	Size of deformation
	*ROI*

#### Regions of Interest

Four regions of interest (ROIs) are selected from sensory cortex, anterior cingulate cortex, the precuneus, and superior-temporal cortex to represent areas of varying structural complexity (Table 4). In this report, for simplicity of illustrations, we have used cubic ROIs but any shape can be selected, with different dimensions, degrees of deformation, and applied to any part of the brain image.

**TABLE 4:** Information about the selected ROIs and applied deformations.

	World coordinates (x,y,z)	Location	Contraction ratio (%)	ROI size (mm)
**ROI-1**	16, −6, 77	Sensory area	Volume: 5, 20, 30, and 40%.	2 mm, 5 mm and 10 mm
**ROI-2**	13, 49, 13	ACC	One dimension: 1.67, 7.17, 11.21, and 15.66%	
**ROI-3**	2, −58, 40	Precuneus		
**ROI-4**	70, −11, 25	Superior Temporal area		

*A given coordinate represents the top/left/back point of the ROI bounding box.*

Cubic ROIs are defined by stereotaxic coordinates at the top, left, and back of the bounding box which extends by the size of each dimension. A tolerance area is set to make sure the deformation field does not alter the image outside the ROI mask. To verify this, we compare the unaltered and the deformed MRIs to assure that only voxels inside the ROI have changed. The center of the ROI cubes are defined as the center of the deformation.

The simulation toolkit allows one to configure the shape, location, and degree of deformation (% of contraction/expansion) of the simulated lesions and can be used for other types of deformations. In this study, the sizes of the cubic ROIs were 2, 5, and 10 mm isotropic, each expressed at 5, 20, 30, and 40% of contraction in 3D volume (creating 1.67, 7.17, 11.21, and 15.66% in each dimension).

The resulting subtle, localized changes in the cortex in the four ROIs used in the present work is shown in Supplementary Material. A video of these ROIs can be accessed at: https://github.com/aces/simulation_toolkit_statistics.

#### Thickness Metrics

Cortical thickness measures can be derived from a variety of methods (Fischl and Dale, 2000; Lerch et al., 2005). We used CIVET’s recommended cortical metric, tlaplace, which represent the linear piecewise distance between the two ends of a non-straight line connecting the WM and GM boundaries (Jones et al., 2000); and FreeSurfer’s thickness metric that measures the distance to the closest point between nodes on two opposite surfaces, and averages the two values (Fischl and Dale, 2000).

#### Blurring Kernels

FWHM (full width at half maximum) represents the amount of gaussian smoothing applied to the distance/thickness metrics.

#### Neuroimaging Pipelines

We use two popular methods of the fully automated cortical thickness estimation, CIVET (MacDonald et al., 2000; Zijdenbos et al., 2002; Tohka et al., 2004; Kim et al., 2005; Lerch et al., 2005; Lee J. et al., 2006; Lee J. K. et al., 2006; Lepage et al., 2020) and FreeSurfer (Dale and Sereno, 1993; Dale et al., 1999; Fischl et al.,1999a,b, 2001, 2002; Fischl and Dale, 2000; Fischl, 2012), to illustrate the application of our proposed platform in comparing either different pipelines or different versions of the same pipeline. Key among our objectives is to evaluate the ability of each pipeline to detect the artificial lesions described above by measuring specificity and sensitivity of detection. These two neuroimaging pipelines have different approaches (Redolfi et al., 2015) and different processing stages to estimate cortical thickness and thus will have different sensitivities to local lesions. This highlights the interest of the toolkit we are presenting here to analyze the impact of any changes along the many stages of each method and differences in sensitivity and specificity between pipelines. These pipelines, as well as the simulation toolkit used in this study (SimDeformation) are available through CBRAIN platform (https://www.cbrain.ca) (Sherif et al., 2014).

#### Deformation Ratio

**FIGURE 2:**
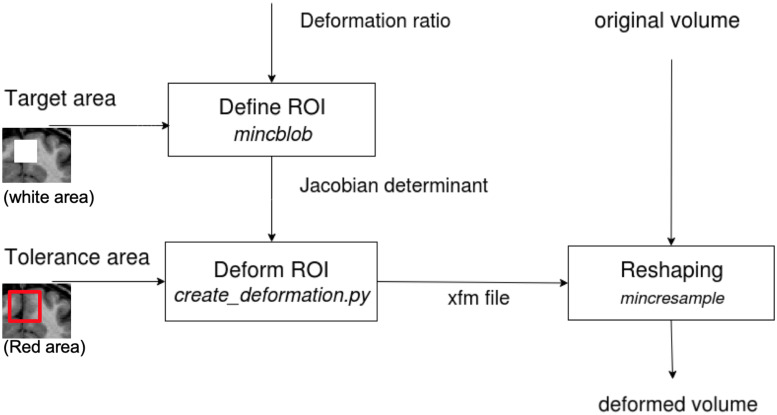
The diagram shows the tissue deformation steps of the brain volume. The input and output volumes are in MINC format (for which DICOM and NIfTI converters are available). The determinant matrix shows the painted GM in the box to be deformed. In our tests, we deformed the whole box in stereotaxic space, since our data are from different subjects or are repeated scans of one subject and have different GM layer structure.

The deformation method is adopted from Van Eede’s simulation platform that generates lesions in the mouse brain (Van Eede et al., 2013) by deforming the structures at a given coordinate-based Region of Interest (ROI). Figure 2 illustrates the stages of this transformation:

• First, a desired Jacobian determinant, with a value below 1 for contraction and above 1 for expansion, is applied to voxels inside the ROI. Voxels outside the ROI have a determinant equal to one (no change).
• Second, a tolerance area (a “pad” of voxels outside of the brain or a ring around the ROI) is specified to restrict the deformations to the desired area and leave the rest of the image unaffected. This is required so that the deformation field can be applied correctly (in the following step) to the ROI without making any unwanted deformation beyond its boundaries.
• Third, an iterative algorithm creates a deformation field from the input of the two previous steps (Jacobian determinant and tolerance area) for subsequent application to the original MRI volume (for more details, please see the Github, https://github.com/Mouse-Imaging-Centre/generate_deformation_fields).

In reality, lesions in the brain induce a measurable effect on the registration of a brain image to the stereotaxic template (Ceccarelli et al., 2012; de Jong et al., 2017). For this reason, and in order to restrict the scope of simulation to surface extraction, we performed the lesion simulation on T1W images that were already registered into the MNI152 stereotaxic space. Using this approach, we also removed the uncertainties that would have emerged from a human anatomist having to select the brain regions where the lesion was positioned. To do so, we defined an ROI at a given stereotaxic location and defined a lesion size by setting the dimensions and the shape of the lesion around that ROI. In principle, the size and shape of the lesion can be modified by setting different values for x, y and z, and choosing different mask shapes. However, for the purpose of this report, we were not interested in identifying the sensitivity of the pipelines to shape variations, therefore we chose isotropic deformations within cubical or ellipsoidal masks. This ensured that the lesion would be applied to the same brain region in all MRIs included in the analysis, assisting us in addressing the objective of pipeline reproducibility. It should be noted that the original and the deformed scans were in an identical MNI152 space and served as inputs to both CIVET and FreeSurfer for all simulations.

### Statistical Analysis

Statistical analyses and visualizations were performed using Surfstat (Worsley et al., 2009). Surfstat is a flexible MATLAB toolbox designed to analyse both surface and volumetric data, with mixed effects models and correction for multiple comparisons by way of random field theory^3^.

Cortical thickness estimations of MRIs with simulated lesions and their original, unaltered counterparts were compared (see Figure 1). The IBIS-Phantom and ICBM datasets allowed us to examine within-subject and population-level effects, respectively (see below), and, in the latter case, both dependent and independent tests were done for FreeSurfer (6.0 and 5.3) and CIVET 2.1.1.

#### Coefficient of Variation

Coefficient of variation: The CoV measures the test-retest reliability of a parameter. It is the standard deviation proportional to the mean of the variable. We used this metric for within-subject tests (IBIS-Phantom dataset). Since anatomical variability is negligible in these repeated scans of a single participant, the CoV should be highly specific to the variability introduced by the simulated lesion. The CoV is calculated at each surface vertex across all scans.

#### Statistical Parametric Mapping

Statistical parametric maps resulting from a Generalized Linear Model (GLM) comparing deformed and unaltered datasets illustrate the ability of a given pipeline (in our case, FreeSurfer and CIVET) to recover the simulated lesions. These include *t*-value and *p*-value maps over the brain surface.

M⁢o⁢d⁢e⁢l=1+D⁢e⁢f⁢o⁢r⁢m⁢a⁢t⁢i⁢o⁢n⁢(r⁢a⁢t⁢i⁢o)+S⁢u⁢b⁢j⁢e⁢c⁢t,

and the difference between “unaltered” and “deformed” groups are studied.

#### Sensitivity and Specificity of Lesion Detection

Metrics of sensitivity and specificity are calculated as at group level:

$$S\,e\,n\,s\,i\,t\,i\,v\,i\,t\,y=\frac{T\,P}{\left(T\,P+F\,N\right)},$$

$$S\,p\,e\,c\,i\,f\,i\,c\,i\,t\,y=\frac{T\,N}{\left(T\,N+F\,P\right)};$$

with *TP* (true positive, the predicted lesion corresponds to an actual lesion), *TN* (true negative, where no lesion is predicted, and there is no actual lesion), *FP* (false positive, if a lesion is predicted where there is no actual lesion) and *FN* (false negative, if no lesion is detected where there is an actual lesion).

The distribution of *t*-values obtained from Statistical Parametric Mapping (SPM) over all vertices of the mid-surface was plotted against the Euclidean distance of the vertex to the center of the ROI in scatter grams.

## Results

### Validation of the Simulation Method

**FIGURE 3:**
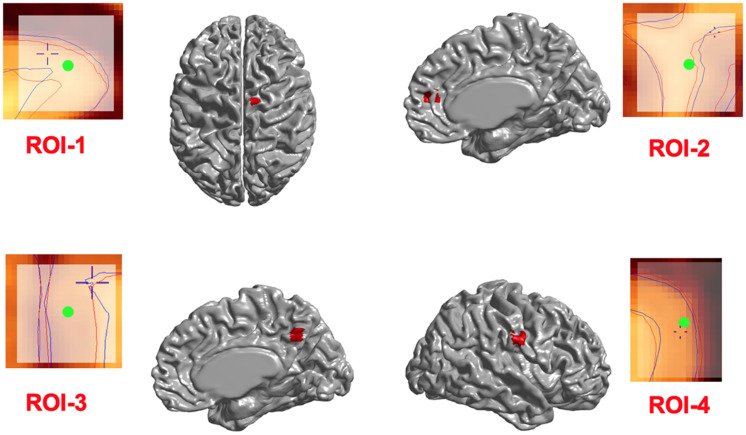
Induced deformations (simulated lesions). Selected ROIs on the mid-surface (shown in red) and the surface extraction before (blue lines) and after (red lines) deformation in Civet 2.1.1. The Center of ROIs are shown in green.

We first investigated whether the regional deformation did induce changes in cortical morphometry. Figure 3 illustrates the changes in cortical surfaces caused by lesions induced in 4 different parts of the brain, and subtraction images that illustrate the focal effects of lesion on the GM surfaces.

**FIGURE 4:**
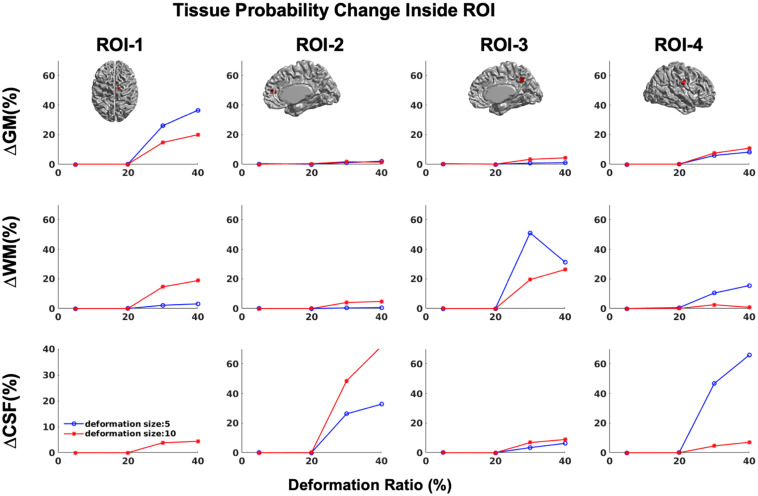
Tissue probability changes (%) versus deformation ratio inside the mask for CIVET 2.1.1. Note that all standard deviations are on the order of 10-3 and are not visible in the plots. (Top) Changes in GM tissue probability versus Volume changes. (Middle) Changes in WM tissue probability versus Volume changes. (Bottom) Changes in CSF tissue probability versus Volume changes.

**FIGURE 5:**
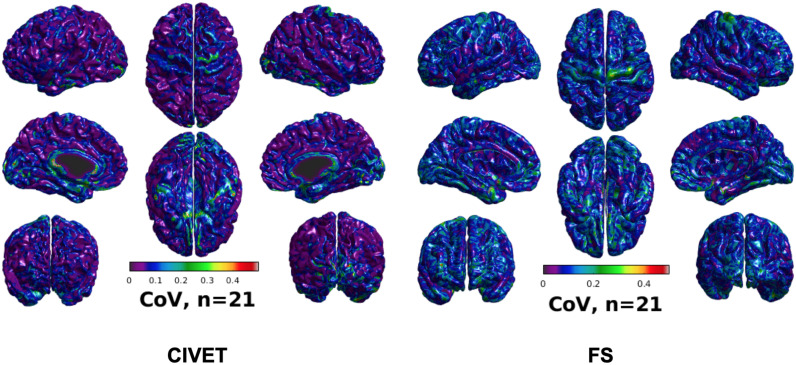
CoV of cortical thickness for IBIS-Phantom brain scans (*N* = 21), CIVET 2.1.1:*tlaplace* and FreeSurfer 6.0.

**FIGURE 6:**
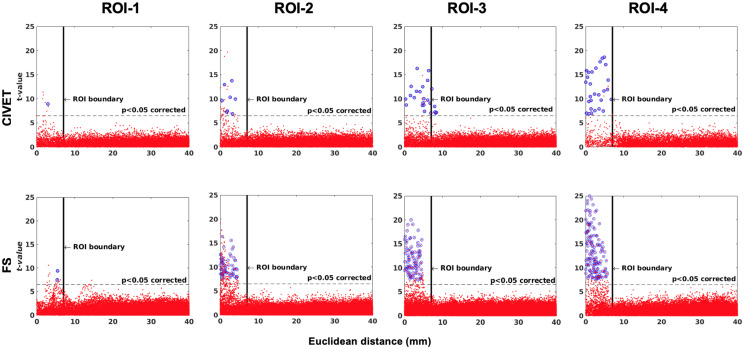
The effect of ROI location (ROI size = 10 mm, FWHM = 0 mm, 15.66% contraction in each direction for four defined cubic ROIs. IBIS-Phantom, *N* = 21), CIVET 2.1.1:*tlaplace* and FreeSurfer 6.0. Euclidean distance is measured between the distortion center to each vertex of the mid surface.

In addition, in order to ensure that simulations did not cause unexpected changes in tissue classification outside the selected ROIs, we examined changes in the ratios of gray, white, and CSF tissue classes inside and outside each ROI for IBIS-phantom (Figures 4–6). The changes outside the mask were negligible for all tissue classes and ROIs (less than 0.2% change in tissue probability). These tests illustrate that the extent of changes inside the masks depends on the location of the ROI and the proportion of each tissue class (GM/WM/CSF) inside the ROI. The discussion of the regional sensitivity of this simulation methods is outside the scope of this current report. However, we can see that the greatest changes were in ROI-1 for GM, ROI-2 for CSF, ROI-3 for WM, and ROI-4 for GM/CSF. Consequently, the most easily detectable difference in cortical thickness was in ROI-4, followed by ROI-2. In the subsequent analysis, we illustrate between-pipeline differences in detecting these ROIs.

### Experiment 1: Single Subject Study

#### Variability

To investigate intra-subject scan variability, we used the coefficient of variation (CoV) in cortical thickness measures at each vertex of the 21 unaltered IBIS-Phantom scans. As can be seen in Figure 5, CoVs are generally low over most of the brain, indicating high reproducibility of cortical thickness calculations for repeated scans with both CIVET 2.1.1 and FreeSurfer 6.

#### Sensitivity and Specificity

**FIGURE 7:**
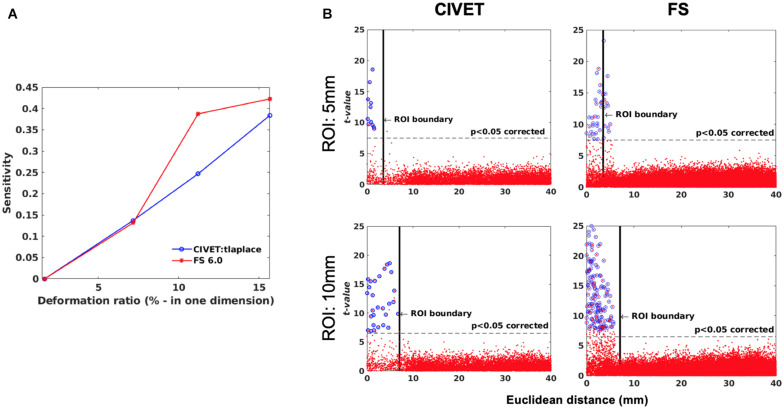
**(A)** Sensitivity versus deformation ratio, IBIS-Phantom. **(B)** Scattergrams of statistically significant vertices. The effect of ROI size (ROI-4, FWHM = 0 mm, 15.66% deformation in each direction), IBIS-Phantom *N* = 21. Higher *t*-values are expected near the deformation point (near zero in the plots). The scattergrams show the vertices that fall within the statistically significant thresholds in blue and the vertical black line illustrates the ROI boundary, CIVET 2.1.1:*tlaplace* and FreeSurfer 6.0. Euclidean distance is measured between the distortion center to each vertex of the mid surface.

**FIGURE 8:**
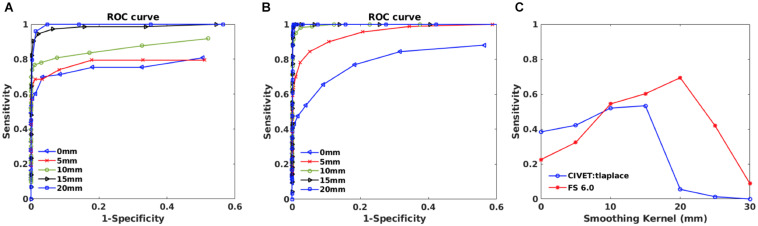
Within subject analysis results for IBIS-Phantom (*N* = 21). **(A)** ROC curves – CIVET 2.1.1. **(B)** ROC Curves - FreeSurfer 6.0. **(C)** Sensitivity and specificity versus smoothing kernel.

Sensitivity and specificity results are plotted in Figures 6–8 for the 21 IBIS-phantom (single-subject) scans. Both FreeSurfer and CIVET are sensitive to the simulated lesions and have high specificity. The sensitivity and specificity are 48.1 and 100% for CIVET 2.1.1 and 60.2 and 100% for FreeSurfer 6.0, on average across ROIs. This means that both pipelines detected some, but not all, of the vertices in a given ROI and, thus, sensitivity is less than 100%. Neither pipeline generated false positives. We should note that the shape of the lesion had some effect on the extent of accuracy. In Supplementary Figure 3, we have compared the effect of choosing an ellipsoidal lesion (which is more realistic) versus a cubic ROI (which induces a stronger deformation) and as can be seen, the more realistic lesion increases the specificity.

The threshold of statistical significance is set at a Bonferroni-corrected *p* < 0.05 (*T*-value >7.5). The *t*-value scattergrams (Figure 6) illustrate the ability of both methods to detect the deformations in the four ROIs. Vertices that fall within the statistically significant thresholds are in blue and the vertical black line illustrates the ROI boundary. Therefore, the blue dots in the scattergrams have a *t*-value (*y*-axis) that corresponds to a significant *p*-value. The *x*-axis shows Euclidean distance of a point on the surface toward the center point of the ROI that was deformed.

#### Effect of Lesion Size and Deformation Ratio on Accuracy of Lesion Detection

Sensitivity and specificity are calculated for ROI-4 (at the size of 10 mm and FWHM of 0 mm) for different deformation ratios (Figure 7A). ROI-4 is chosen based on the changes in tissue probability calculated in Section “Validation of the Simulation Method,” where this ROI has the higher changes in the cortical area. As expected, sensitivity increases with deformation ratio and 7% contraction in one dimension (0.07 mm change) was the detection threshold for all measures and methods within this single-subject repeated-scan sample. CIVET and Freesurfer both have a specificity of 1 in all tests. Statistical significance is set to Bonferroni-corrected *p* < 0.05 (*t*-value >7.5).

Figure 7B illustrates the distribution of all vertices of the mid surface, with their *t*-values plotted against Euclidean distance from the deformation core point. We expect to have higher *t*-values near the deformation point (near zero in the plots). Vertices that fall within the statistically significant thresholds are in blue and the vertical black line illustrates the ROI boundary. We can observe that neither CIVET nor FreesSurfer has false positives distal to the lesion. Also, sensitivity increases with increasing lesion size for both CIVET 2.1.1 and FreeSurfer 6.0. Note that the number of vertices in FreeSurfer surfaces is four times that of CIVET 2.1.1, which accounts for the greater number of blue points for FreeSurfer in Figure 7B.

#### Effect of Blurring Kernel on the Accuracy of Lesion Detection

ROC curves are plotted in Figures 8A,B for different smoothing kernels for both pipelines. Plots show that applying smoothing increases the sensitivity and specificity for CIVET 2.1.1 and FreeSurfer 6.0.

Sensitivity and specificity versus smoothing kernel size are depicted in Figure 8C for the selected thresholds (*p* < 0.05 and *T*-value >7.5) and indicates that there is a peak in sensitivity for each method, with 10 mm being best for CIVET 2.1.1 and FreeSurfer 6.0 has maximum sensitivity at 20 mm and Specificity is equal to 1 for both CIVET and FreeSurfer.

### Population Simulation

#### Experiment 2 (Pre- and Post-lesion)

**FIGURE 9:**
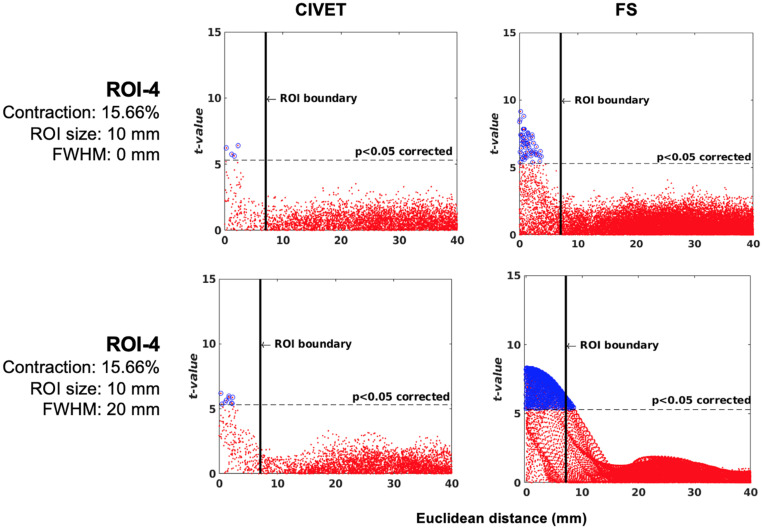
Scattergrams of the statistically significant vertices for the ICBM dataset (*N* = 152), dependent tests. Higher *t*-values are expected near the deformation point (near zero in the plots). Vertices that fall within the statistically significant thresholds are in blue and the vertical black line illustrates the ROI boundary. The contraction ratios show the amount of deformation for a single dimension, CIVET 2.1.1:*tlaplace* and FreeSurfer 6.0. Euclidean distance is measured between the distortion center to each vertex of the mid surface.

These analyses used the 152 MNI-ICBM individual MRIs, unaltered and deformed and using the same four ROIs, as per our method. Figure 9 displays *t*-values for all vertices on the mid surface versus their Euclidean distance from the center of the ROI. Statistically significant vertices are in shown blue and the thresholds are Bonferroni-corrected at *p* < 0.05 and *t* > 7.5 (DoF = 151, *t*(*df* = 151) > 7.5, *p* > 0.05). The ROI boundary is indicated by the vertical black line.

Neither method had false positives and their specificity was equal to 1. Furthermore, both methods were sensitive to local changes and, as was the case for IBIS-Phantom (single-subject scans, Section “Experiment 1: Single Subject Study”), the highest sensitivity was to ROI-4. Therefore, sensitivity was again dependent on the location of the ROI.

The tolerance box size and deformation ratio to which the techniques were sensitive was 10 mm and 11.21% (in each dimension). This means that, for the ROIs tested here and in a population sample of 152 individuals, the methods are sensitive to a 0.1121 mm change in cortical thickness. Furthermore, the number of true positives increased by increasing the smoothing kernel.

#### Experiment 3 (Group 1 – Group 2)

**FIGURE 10:**
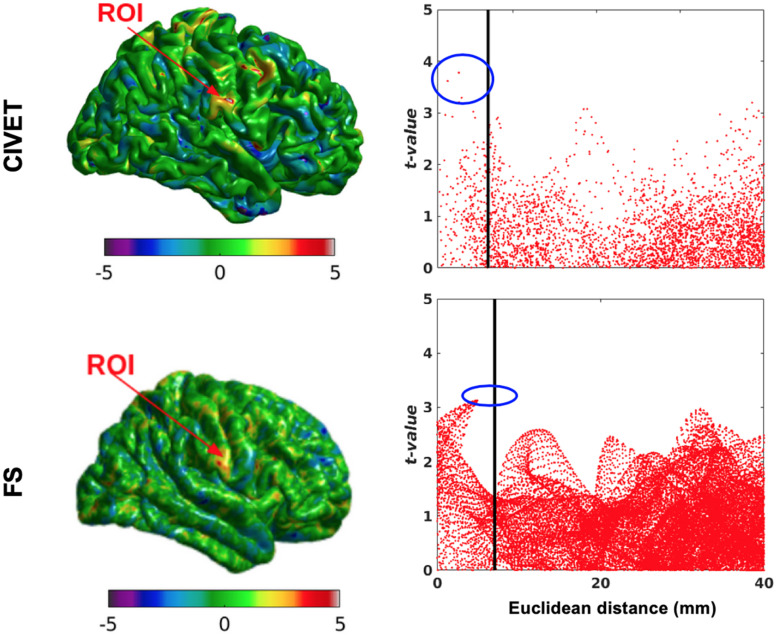
Surface t-maps and scattergrams of the statistically significant vertices for the ICBM dataset, independent tests (*N* = 76). ROI-4, 15.66% contraction (one dimension), FWHM: 10 mm, ROI size: 10 mm, CIVET 2.1.1:*tlaplace* and FreeSurfer 6.0. Euclidean distance is measured between the distortion center to each vertex of the mid surface.

We randomly assigned half (76) of the 152 subjects to an “unaltered” group and the other half to the “deformed” group (Table 4), whose images were altered with simulated lesions with the same coordinates as in Table 2, as per our method. This independent test shows lower *t*-values compared to dependent tests and therefore the sensitivity is lower for both pipelines at the statistical map level, while the specificity is still equal to 1. However, Figure 10 shows there are higher *t*-values inside the ROI than outside. The thresholds are Bonferroni-corrected to *p* < 0.05 and *t* > 7.5 and the DoF is 150 [*t*(*df* = 150) > 7.5, *p* > 0.05].

### Software Version Comparison

**FIGURE 11:**
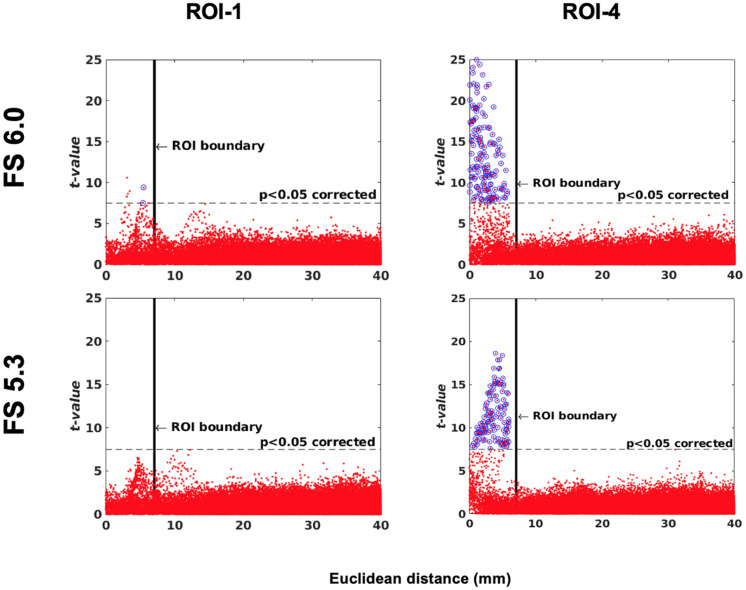
Software version comparison: simulation tests between versions 5.3 and 6.0 of FreeSurfer. Scattergrams of the statistically significant vertices for IBIS-Phantom *N* = 21. Higher *t*-values are expected near the deformation point (near zero in the plots). Vertices that fall within the statistically significant thresholds are in blue and the vertical black line illustrates the ROI boundary. The effect of ROI location (ROI size = 10 mm, FWHM = 0 mm, 15.66% deformation in each direction). Euclidean distance is measured between the distortion center to each vertex of the mid surface.

One of the main applications of this simulation toolkit is to study and document changes in performance between versions of any one neuroimaging pipeline. Here, we show a simple use case for this toolkit by illustrating the changes in sensitivity and specificity between two versions of the same pipeline, FreeSurfer: 6.0 and 5.3 using a single-subject (IBIS-Phantom) dataset. The scattergrams in Figure 11 show that the newer version has higher sensitivity (more true positives) in all the tested ROIs, which justifies using newer version of the tool in future studies.

## Discussion

The goal of this work is to propose a general simulation framework that can be used to study and document differences between any two automated pipelines or, versions of the same pipeline. It can be used to investigate the sensitivity and specificity in response to small induced perturbations in brain images, providing coefficient of variance and statistical significance maps for either within- or between-subject tests.

### Sensitivity and Specificity

With simulated lesions in four ROIs, our results illustrate the high specificity and sensitivity of both CIVET and FreeSurfer. Neither pipeline yields false positives in any of the tested ROIs, irrespective of deformation ratio or blurring kernel size.

Sensitivity was dependent on the location and degree of deformation. The partial volume effects vary in different regions thus introduce variations in the tissue properties of the areas at which the deformation is applied. While such effects in a simulation platform are caveats, they nevertheless serve to illustrate whether corticometry pipelines are sensitive to such spurious alterations. The cortical thickness pipelines such as CIVET or FreeSurfer involve very complex concatenations of processing steps (e.g., registration, tissue classification, surface extraction, etc.) each of which will yield somewhat different results with even small perturbations to an image.

While our toolkit is not suitable for a head-to-head comparison of different stages of computation involved in cortical thickness measurement, it does provide simple (even though crude) evidence about the validity of a pipeline. For example, to detect false positives in an un-lesioned area in a simulation study can help avoid erroneous interpretations of data processed through faulty pipelines. Among regions with simulated lesions in this study, both pipelines were maximally sensitive to ROI-4, a region which had proportionally accurate simulated changes in the GM volume. This suggests that regional variations in tissue partial volume may influence the accuracy and sensitivity of corticometry pipelines to detecting real abnormalities. This simulation toolkit allows us to investigate such questions, for example by creating a statistical parametric map to assess the detectability of simulated cortical lesions across the cortical mid-surface.

### The Effect of Deformation Ratio and Size of the Lesion

In this study, there were no false positives and, therefore, specificity was equal to 1 in all tests. The changes (deformation/shrinkage) should be at least 0.35 mm [0.07^∗^5 (mm-size of ROI)] for within-subject tests, and 1.12 mm [0.11^∗^10 (mm-size of ROI)] for population-level tests, in order for them to be statistically detectable.

### Blurring Effects

Blurring is often used to increase detectability of lesions that are slightly offset from one another. We found that the 5 and 10 mm FWHM are the best choices and match the ROI sizes in Section “Results and Discussion”. This is expected based on Lerch and Evans (2005): if there is prior information of the induced lesion, the smoothing kernel FWHM should match the size of the simulated area. In the case where no prior information is available, FWHM can be estimated according to the number of subjects/scans, where a small number can benefit from increased sensitivity with a larger blurring kernel. On the other hand, as shown in Section “Results and Discussion,” increased smoothing may decrease sensitivity when the number of statistically significant points is small (as was the case for CIVET) or increase, sensitivity at the cost of specificity when there are more statistically significant points (as was the case for FreeSurfer). Normalized standard deviation for different smoothing kernels was calculated for the unaltered images of IBIS-phantom dataset (Supplementary Figure 1) and the results indicate a decline with increasing kernel size. This shows that variability decreases with increasing FWHM and this leads to underestimation of the size of the simulated lesion in CIVET.

Also, in examining the effect of FWHM on cortical thickness measures, we observe a decline of normalized standard deviations with increasing smoothing kernel size for unaltered images (Supplementary Figure 2). This shows that variability decreases with increasing FWHM which, in turn, may lead to underestimation of the size and extent of the simulated lesions.

### Limitations and Future Work

This study introduces a tool that can be used to compare the performance of different automatic cortical thickness estimation pipelines (e.g., CIVET, FreeSurfer) or different versions of a given pipeline using simulated lesions. However, several limitations need to be considered.

First, in the current analysis we only introduced cubical and ellipsoidal lesions in cortical ROIs, and did not experiment with other lesion shapes different types of lesions. However, the deformation toolkit is not limited such lesions, nor to human cortex, and in fact has been used previously to validate the sensitivity of imaging pipelines for detecting subcortical shape variations in rodents (Van Eede et al., 2013). Therefore this methodology is also applicable for testing deformation-based morphometry (DBM).

Second, we selected lesion locations based on our experience that these regions are subject to image processing inaccuracies emerging from high anatomical variability or partial volume effects. In future work, the sensitivity and specificity probability map could be calculated for the entire brain by selecting more realistic lesions across various other parts of the brain. However, it should be noted that given the anatomical features of the cerebellum, the current tool may not be suitable for simulating cerebellar lesions.

Third, we have introduced the lesions in the brains of healthy adults, in stereotaxic space and have used these spatially normalized images as input to the corticometry pipelines. The advantage of this approach is that it isolates the effect of the simulated lesions on the surface extraction and corticometry algorithms; however, as a result it does not fully account for variations that may emerge in the registration of atrophied, or lesioned brains to normal templates, thus there is room for extending the current simulation platform.

Finally, to evaluate the performance of pipelines against each other is outside the scope of this report. Our results illustrate that with the accuracy in both pipelines is 100%, the larger number of vertices used to tesselate the surface in FreeSurfer increases the degrees of freedom and offers a moderate advantage in sensitivity (62%) versus CIVET (48%), however, this comes at the cost of increased computation time. Whether these metrics are consistent across lesions in different parts of the brain remains to be evaluated.

## Conclusion

In this work, we propose a general simulation platform to comparatively evaluate the sensitivity and specificity of different neuroimaging pipelines to simulated lesions in MRIs. We used two datasets (IBIS-Phantom and ICBM, for intra- and inter-subject analysis, respectively) and deformed to varying and controlled degrees small, coordinate-based areas of T1-weighted images in stereotaxic space. We then used two commonly-used pipelines (CIVET and FreeSurfer) to estimate cortical thickness from unaltered and deformed volumes and analyzed the accuracy of different thickness metrics and blurring kernels. This simulation toolkit can be used to simulate lesions in a controlled manner and statistically analyze their effects on cortical thickness estimation.

## Data Availability Statement

Data used in this study is publicly available. The ICBM data set can be obtained from https://ida.loni.usc.edu/collaboration/access/appLicense.jsp. The IBIS phantom data (*N* = 21) is available for limited use on CBRAIN platform (https://portal.cbrain.mcgill.ca/). All scripts and deformed regions, as well as statistical tests can be obtained via https://github.com/aces/simulation_toolkit_singularity.

## Ethics Statement

Ethical review and approval was not required for the study on human participants in accordance with the local legislation and institutional requirements. Written informed consent for participation was not required for this study in accordance with the national legislation and the institutional requirements. Written informed consent was obtained from the individual(s) for the publication of any potentially identifiable images or data included in this article.

## Author Contributions

MO and NK-M contributed equally to the study design and analyses. MO, AR, and PB analyzed the data. CL, LL, MV, SJ, RV, PR, and AZ developed image processing and computational tools for the study. MO, NK-M, and PB wrote the manuscript. All authors reviewed and contributed to the final manuscript. RA and AE supported the entire team and the study. All authors contributed to the article and approved the submitted version.

## Conflict of Interest

The authors declare that the research was conducted in the absence of any commercial or financial relationships that could be construed as a potential conflict of interest.

## Footnotes

1 The Infant Brain Imaging Study (IBIS) Network is an NIH funded Autism Center of Excellence project and consists of a consortium of 8 universities in the U.S. and Canada. Data used in this study is from a traveling phantom, and is made available for this specific study by approval from PIs of the IBIS Network. https://autismbabybrain.com

2 https://ida.loni.usc.edu/collaboration/access/appLicense.jsp

3 https://www.math.mcgill.ca/keith/surfstat/, Information about statistic.

## Supplementary Material

Supplementary Figure 1 Normalized standard deviation versus blurring kernel. IBIS-Phantom, original scans, *N* = 21, CIVET 2.1.1:*tlaplace* and FreeSurfer 6.0 and 5.3.

Supplementary Figure 2 Within subject analysis results. **(A)** *ROC* curves – Blurring kernel: 0 mm, **(B)** *ROC* curves – Blurring kernel: 15 mm, **(C)** Sensitivity and specificity versus smoothing kernel. IBIS-Phantom (*N* = 21), CIVET 2.1.1:*tlaplace* and FreeSurfer 6.0.

Supplementary Figure 3 Effects of lesion shape on the accuracy of lesion detection and statistical detectability. Comparisons are performed in ROI-4 (FWHM = 0 mm, 15.66% deformation in each direction) in the IBIS-Phantom (*N* = 21, single subject). Scattergrams illustrate the Euclidean distance of detected lesions from the ROI’s coordinate. Euclidean distance is measured between the distortion center to each vertex of the mid surface. The size of the cubic ROI is 10 mm in each direction, and the radius of the ellipsoidal ROI is 5 mm × 5 mm × 9 mm (x, y, and z directions, respectively), so the volumes of the deformation area are almost the same for both cubic and ellipsoid ROIs. Higher *t*-values are expected near the deformation point (near zero on the *x*-axis). The scattergrams show the vertices that fall within the statistically significant thresholds in blue and the vertical black line illustrates the ROI boundary, CIVET 2.1.1:tlaplace and FreeSurfer 6.0.
