# Supplementary material for: A Simulation Toolkit for Testing the Sensitivity and Accuracy of Corticometry Pipelines
Source: Front Neuroinform. 2021 Jul 26;15:665560. doi: 10.3389/fninf.2021.665560 (PMC8350777; doi:10.3389/fninf.2021.665560)
Supplement: Supplementary Figure 1 — Normalized standard deviation versus blurring kernel. IBIS-Phantom, original scans, N = 21, CIVET 2.1.1:tlaplace and FreeSurfer 6.0 and 5.3. [file Data_Sheet_1.docx]

***Supplementary Material***

# Variability

We expect to have regional variations across the brain cortex (Lerch and Evans, 2005) for the unaltered MRIs. The vertex-based Coefficients of Variation (CoV) of the cortical thickness are studied for IBIS-Phantom dataset in section 3.2, where there are 21 scans of one subject. The CoV maps over the brain show symmetric shape for both pipelines and the CoV values are low all over the brain. However, the structure of CoV is different for pipelines.

Here, the standard deviation of the measured thickness is calculated at each vertex for both the repeated scans of one subject (IBIS-phantom) and the ICBM population and are shown in Supplementary Table 1. For CIVET 2.1.1, tlink has the highest and tfs has the lowest standard deviations. All metrics show small CoV values all over the brain surface, while CIVET has lower CoVs. The mean thickness is differing for different metrics, since each meter has special description. By normalizing the standard deviation, still tlink has the worst performance for CIVET and FreeSurfer 6.0 has improved from FreeSurfer 5.3. All of these is correct for both 21 repeated scans and for 152 different subjects. The standard deviations are higher for ICBM dataset for all pipelines, which is expected due to interindividual variablity.

| **Supplementary Table 1.** Mean and Standard deviations of cortical thickness for different metrics. | | | | | | | | | |
| --- | --- | --- | --- | --- | --- | --- | --- | --- | --- |
|  |  | **21 scans of the same subject**  **(IBIS-Phantom)** | | | | **152 normal subjects**  **(ICBM)** | | | |
|  |  | Mean | STD | Nrm. SD |  | Mean | STD | Nrm. SD |  |
| **CIVET 2.1.1** | Tlaplace | 3.27 | 0.26 | 0.08 |  | 3.27 | 0.53 | 0.16 |  |
|  | Tlink | 3.64 | 0.34 | 0.09 |  | 3.65 | 0.72 | 0.20 |  |
|  | Tfs | 3.03 | 0.21 | 0.07 |  | 2.98 | 0.43 | 0.14 |  |
| **FS 5.3** | | 2.51 | 0.32 | 0.13 |  | 2.48 | 0.63 | 0.25 |  |
| **FS 6.0** | | 2.43 | 0.25 | 0.10 |  | 2.43 | 0.53 | 0.22 |  |

The normalized standard deviation is calculated for different smoothing kernel values and are plotted in Supplementary Figure 1. Results indicate the decline of normalized standard deviations by increasing the smoothing kernel increase.

**
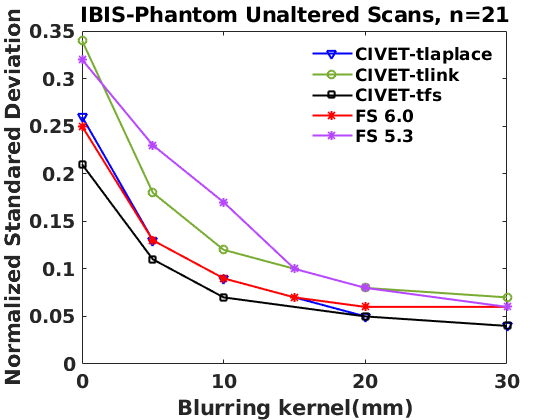
**

**Supplementary Figure 1.** Normalized standard deviation versus blurring kernel. IBIS-Phantom, original scans, n=21, CIVET 2.1.1:*tlaplace* and FreeSurfer 6.0 and 5.3..

# Effect of Blurring Kernel on the Accuracy of Lesion Detection

The sensitivity versus specificity curves are plotted in Supplementary Figure 2.a and 2.b for two different smoothing kernels (0mm and 15mm). As it is seen from the plots, *tlaplace* has the best results for CIVET 2.1.1. The specificity is high for all the metrics for CIVET and both versions of FreeSurfer pipelines and applying the smoothing increases the sensitivity for both pipelines. It means that there are quite a high number of statistically significant vertices in both methods so that this number increases by the smoothing. The sensitivity versus smoothing kernel is depicted in Supplementary Figure 2.c and it indicates that there is a peak for each method, where 10mm is the best for all methods and metrics except FS 6.0 which has the maximum sensitivity at 20mm.

| 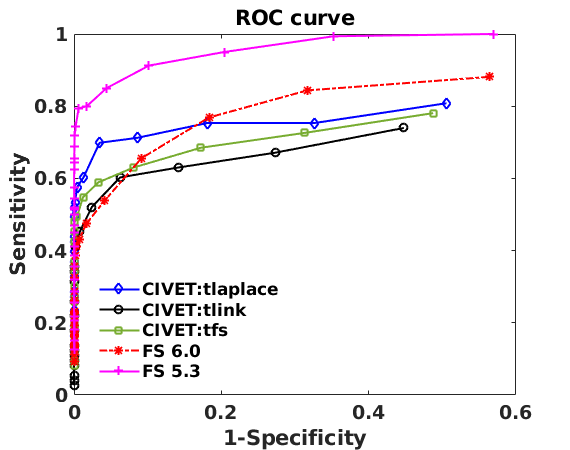 | 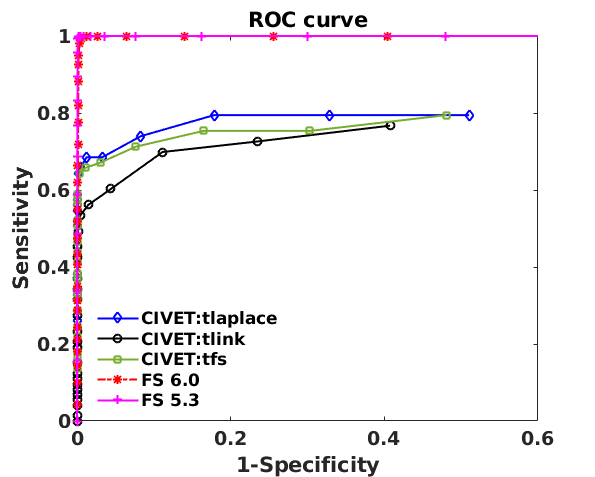 |
| --- | --- |
| **(a)** | **(b)** |
| 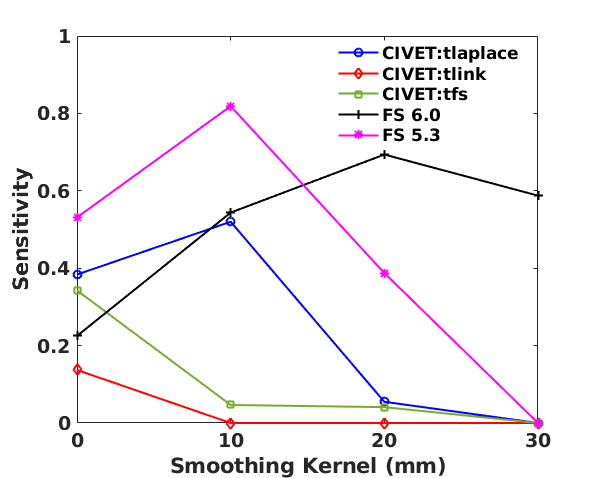 | |
| **(c)** | |
| **Supplementary Figure 2.** Within subject analysis results. (a) *ROC* curves – Blurring kernel: 0*mm,* (b) *ROC* curves – Blurring kernel: 15*mm,* (c) Sensitivity and specificity versus smoothing kernel. IBIS-Phantom (N=21), CIVET 2.1.1:*tlaplace* and FreeSurfer 6.0.  **3. Effect of Lesion Shape on Statistical Parametric Mapping**  For simplicity, we chose cubic lesions for testing the simulation pipeline, but in reality, lesion shapes are not cubical. Therefore we also created an ellipsoidal lesion along the cortical curvature to examine the effects of lesion shape on the accuracy of lesion detection and statistical detectability. Comparisons are performed in ROI-4 (FWHM=0mm, 15.66% deformation in each direction) in the IBIS-Phantom (N=21, single subject). Scattergrams illustrate the Euclidean distance of detected lesions from the ROI's coordinate. Euclidean distance is measured between the distortion centre to each vertex of the mid surface. The size of the cubic ROI is 10 mm in each direction, and the radius of the ellipsoidal ROI is 5x5x9mm (x, y and z directions, respectively), so the volumes of the deformation area are almost the same for both cubic and ellipsoid ROIs. Higher t-values are expected near the deformation point (near zero on the x-axis). The scattergrams show the vertices that fall within the statistically significant thresholds in blue and the vertical black line illustrates the ROI boundary, CIVET 2.1.1:tlaplace and FreeSurfer 6.0.  **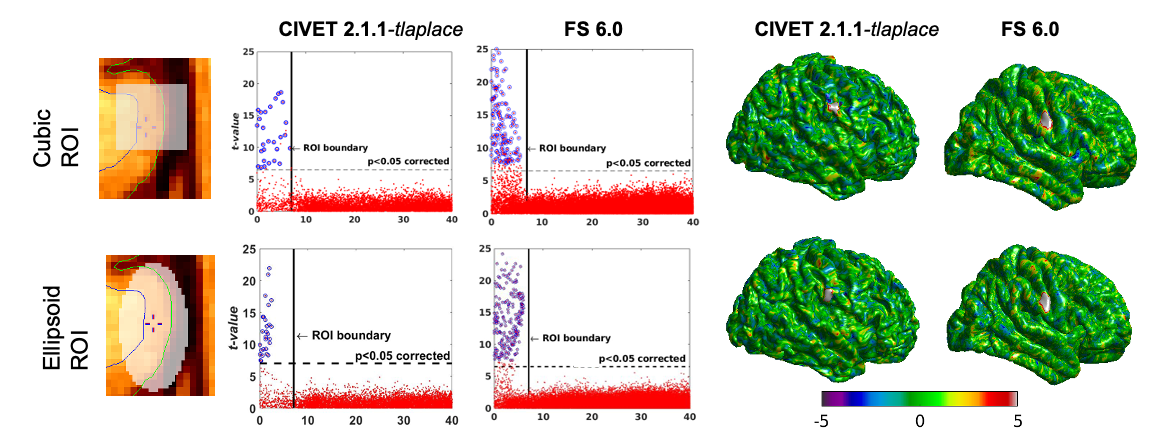** | |

**Supplementary Figure 3.** Effect of lesion shape on lesion detection accuracy. To choose a more realistic lesion shape (ellipsoidal versus cubic) increases the accuracy of detecting the lesions, especially for CIVET.
